# Effects of Temperature and Salinity on Ovarian Development and Differences in Energy Metabolism Between Reproduction and Growth During Ovarian Development in the *Lateolabrax maculatus*

**DOI:** 10.3390/ijms26178295

**Published:** 2025-08-27

**Authors:** Yangtao Peng, Lulu Yan, Chao Zhao, Bo Zhang, Bo Zhang, Lihua Qiu

**Affiliations:** 1 State Key Laboratory of Mariculture Biobreeding and Sustainable Goods (BRESG), South China Sea Fisheries Research Institute, Chinese Academy of Fishery Sciences, Guangzhou 510300, China; 2 College of Aqua-Life Science and Technology, Shanghai Ocean University, Shanghai 201306, China; 3 Key Laboratory of Aquatic Genomics, Ministry of Agriculture and Rural Affairs, Chinese Academy of Fishery Science, Beijing 100141, China; 4 Sanya Tropical Fisheries Research Institute, Sanya 572000, China

**Keywords:** ovarian development, sex hormone, temperature, salinity, energy metabolism

## Abstract

Fish reproduction requires suitable salinity and temperature, as well as sufficient energy. This study investigated temperature and salinity effects on ovarian development of *Lateolabrax maculatus* and energy metabolism differences between reproduction and growth. Two salinities (4‰ and 30‰) and temperatures (18 ± 1 °C and 30 ± 1 °C) formed four treatments: SWNT (30‰, 30 ± 1 °C), SWLT (30‰, 18 ± 1 °C), FWLT (4‰, 18 ± 1 °C), and FWNT (4‰, 30 ± 1 °C). GSI and sex hormones (FSH, LH, E2, and 17α,20β-DHP) were measured. Transcriptome analysis explored how temperature and salinity regulate ovarian development in *L. maculatus*, while integrated transcriptomic and targeted energy metabolomic analyses revealed energy metabolism differences between ovary and muscle during this process. The results showed that low salinity (4‰) and low temperature (18 ± 1 °C) synergistically promoted ovarian development in the FWLT group, as indicated by a significant increase in GSI and elevated levels of key sex hormones (FSH, LH, E2, and 17α,20β-DHP). Transcriptome analysis showed that low temperature activated pathways involved in steroidogenesis, oocyte maturation, and meiosis, and genes such as *ADCY6*, *PRKACB*, *CPEB4*, *FZD7-A*, and *CCND2* were significantly upregulated. Salinity changes mainly affected amino acid metabolism, cholesterol metabolism, and the insulin signaling pathway. Genes such as *PCSK9* and *CKM* may regulate ovarian development by regulating hormone synthesis and energy metabolism. Comprehensive transcriptome and metabolome analyses show that glycolysis is downregulated and oxidative phosphorylation is upregulated in the ovary, suggesting that ovarian oogenesis tends to be energized by aerobic metabolism. The TCA cycle may be used more for providing biosynthetic precursors and facilitating the transport of substrates between the mitochondrion and the cytoplasm rather than just as a source of ATP. Muscle tissue relies primarily on glycolysis for rapid energy production and may redistribute energy to the gonads, prioritizing the energy needs of the ovaries and contributing to the dynamic balance between reproduction and growth. This study provides insights into the molecular mechanisms of how environmental factors regulate fish reproduction, providing a theoretical basis and potential molecular targets for the regulation of reproduction and optimization of aquaculture environments.

## 1. Introduction

The *Lateolabrax maculatus* ranks as the third most important marine aquaculture species in China. This species is primarily distributed in the western Pacific Ocean, particularly along the Chinese coastline and in major marine areas, such as the Yellow Sea and the Bohai Sea. Major aquaculture sites include Qingdao, Shidao, Qinhuangdao, and the Zhoushan Islands. Under natural conditions, *L. maculatus* spawns in the fall. The wild *L. maculatus* spawned in coastal waters at 19.7 °C in late October and 17.2 °C in early November after starting vitellogenesis in September at about 24.5 °C and developing third-stage vitellogenic oocytes by early to mid-October.

Fish reproduction is regulated by the endocrine system and by external environmental factors, such as temperature, salinity, photoperiod, water flow, pH, dissolved oxygen, and others. Temperature is one of the main factors affecting fish reproduction, as it alters molecular structures and modulates the rates of biochemical reactions and physiological processes [1]. Thus, temperature can differentially influence the neural and endocrine components of the reproductive axis [2]. Water temperature is known to have a strong effect on the brain–pituitary–gonadal axis (BPG) of teleost fish, thereby controlling their reproductive development [3,4]. Fish require a narrower temperature range for reproduction than for growth [5]. The damaging effects of temperatures outside the optimal range on fish reproduction have been studied in several species [6]. For example, *Acanthochromis polyacanthus* produces smaller eggs and exhibits lower reproductive output at 31.5 °C than at 28.5 °C [7]. F1 generation offspring reared at 31.5 °C also exhibited reduced reproductive output [8]. Female *Salvelinus alpinus* reared at 8 °C exhibited delayed ovulation compared to those at 5 °C, while those reared at 11 °C failed to ovulate entirely [9]. Ovulation in *Oncorhynchus mykiss* [10] and *Salmo salar* [11] is also inhibited at unsuitable breeding temperatures. Reproductive activity in the *Cyprinodon nevadensis* was limited at 18 °C, peaked at 28–30 °C, and declined above 32 °C. At temperatures above 34 °C, *C. nevadensis* exhibited reduced E2 levels, downregulated expression of ovarian steroidogenic enzyme genes, and decreased expression of vitellogenin (Vg) and choriogenin egg envelope protein (Ceep) genes [12]. These findings suggest that abnormal breeding temperatures may impair the function of the HPG axis and reduce reproductive performance. Under natural conditions, *L. maculatus* spawns in the fall. The wild *L. maculatus* spawned in coastal waters at 19.7 °C in late October and 17.2 °C in early November after starting vitellogenesis in September at about 24.5 °C and developing third-stage vitellogenic oocytes by early to mid-October. In female *L. maculatus*, the gonadosomatic index (GSI) increased from 0.54 to 10.1, and serum E2 levels rose from 0.3 to 394.6 pg/mL as the water temperature declined from 26.3 °C to 15.3 °C. However, when the water temperature dropped from 15.3 °C to 13.0 °C, the GSI decreased to 5.0 and serum E2 levels declined to 124.8 pg/mL. The ovaries also showed atretic follicles and began to degenerate [13]. The decrease in sex steroid concentrations at supra-optimal breeding temperatures is partly due to reduced stimulation of the HPG axis, which promotes gonadal steroidogenesis, and partly due to the inhibition of the steroidogenic enzyme aromatase (Cyp19a1a), resulting in suppression of E2 synthesis or activity in the gonads [14]. Temperatures above the optimal range can lead to reduced or inhibited E2 production, resulting in smaller eggs, reduced egg survival [15,16,17], and, in severe cases, inhibition of ovulation [6]. Non-optimal breeding temperatures also disrupt oocyte osmoregulation, leading to reduced levels of phospholipids and free fatty acids [18]. Lipid deposition, including triacylglycerols and phospholipids, is also affected during oocyte development in female *S. alpinus* reared at elevated temperatures [19].

Salinity also influences fish reproduction, with changes in salinity affecting spermatogenesis and testicular homeostasis [20], as well as overall gonadal development [21]. *Psammoperca waigiensis* lives in water bodies with salinities ranging from 10 to 30‰. At 10‰ salinity, egg maturation, and spawning rates are reduced, and fertilized eggs do not hatch properly under the same conditions [22]. This shows that fish reproduction can be negatively affected by unsuitable salinity during the reproductive phase. *S. salar* grows in seawater but reproduces in freshwater—when kept in seawater during the breeding season, ovulation is delayed or impaired [23]. Similar results were observed in *Oncorhynchus kisutch* [24]. Changes in salinity had a greater impact on testicular function than on ovarian development. In *Acanthopagrus butcheri*, plasma 11-kT levels in males and E2 levels in females significantly increased at a salinity of 25‰. Oocytes at various developmental stages were observed at salinities ranging from 5‰ to 35‰, with yolk formation induced across all salinity levels. However, full sperm maturation occurred only at the moderate salinity of 25‰ [25]. *Acanthopagrus schlegeli* can survive in salinities from 5‰ to 45‰, and males require more stringent salinity conditions for complete sexual maturation compared to females. Female *A. schlegeli* had similar oocyte sizes from 5‰ to 45‰ salinity, but female *A. schlegeli* at 35‰ and 45‰ salinity had higher ovulation rates and longer ovulation durations, whereas male *A. schlegeli* at less than 15‰ and 45‰ salinity had lower sperm motility [26].

It has been shown that gonadal development in fish requires substantial energy input. Hepatic metabolomic analyses in *Carassius auratus* revealed that metabolic energy is redistributed across different reproductive stages to support gonadal growth and body mass increase, emphasizing the high energetic demands associated with the production of hundreds of eggs and large quantities of sperm [27]. This underscores the critical importance of energy supply during gonadal development. At the physiological level, fish typically mobilize lipid and protein reserves to meet the energetic demands of reproduction. For example, in *Megalobrama terminalis*, ovarian lipid content progressively increases during reproductive migration, whereas lipid levels in trunk tissues, such as muscle, significantly decline [28]. This reduction in somatic lipid content primarily serves as an energy source for reproductive migration [29,30]. Moreover, studies on *Pampus argenteus* suggest that insulin-like growth factor 1 (IGF1) facilitates yolk formation and spermatogenesis by modulating ovarian energy metabolism [31], further highlighting the role of metabolic regulation in gonadal development. Additional metabolomic studies indicate that energy-related molecules, such as ATP, ADP, and AMP, undergo dynamic fluctuations during reproductive stages. Although conducted on a different species, research on the *Mytilus edulis* demonstrated that these adenylates, acting as cellular “energy currency,” vary significantly during gonadal development and are correlated with reproductive quality [32], supporting the pivotal role of adenylate metabolism in reproduction. From a life-history perspective, fish must balance energy allocation among competing physiological demands, such as growth, migration, and reproduction, to optimize fitness [33]. Females generally invest more energy in gonadal development than males [34], prioritizing reproductive output under limited energy conditions. As a result, many fish exhibit a reproductive-focused metabolic strategy, channeling energy resources toward reproduction. This strategy is particularly evident in migratory species, such as *S. salar*. During migration, *S. salar* ceases feeding, relying entirely on endogenous energy reserves—primarily fat and muscle—to support reproductive processes, while the gonads are spared for successful gamete development. Post-spawning mortality due to energy depletion is common, illustrating the trade-off of somatic energy for reproductive success. The energetic demands are especially acute during vitellogenesis. In *C. auratus*, yolk formation alone requires a substantial metabolic energy investment [27]. Similarly, in *O. mykiss*, the gonadosomatic index (GSI)—the ratio of ovary mass to body mass—increases dramatically from approximately 0.5% to nearly 20% prior to spawning [35], reflecting the massive energy investment required within a short timeframe.

Although temperature effects on fish gonadal development have been extensively studied, the underlying molecular regulatory mechanisms remain unclear and warrant further investigation. Compared to temperature, the regulation of gonadal development by salinity has been less explored, primarily focusing on males, with even fewer studies addressing females. Similarly, the mechanisms through which salinity influences gonadal development remain poorly understood, necessitating further research to elucidate these effects and underlying pathways in fish. Although overall energy allocation trends between growth and reproduction have been preliminarily studied in fish, molecular-level differences in energy metabolism between key tissues involved in growth and reproduction—namely, muscle and gonads—remain unclear. We analyzed changes in gonadal indices and reproduction-related hormone levels in female *L. maculatus* exposed to varying temperatures and salinities. Using transcriptomic technology, we further investigated the effects of these environmental factors on ovarian development by profiling gene expression changes and identifying candidate genes responsive to temperature and salinity during ovarian development. The group exhibiting optimal ovarian development was selected to study differences in energy metabolism between ovary and muscle tissues during the ovarian development period, employing transcriptomic and targeted energy metabolomics analyses. This study will provide valuable insights into how temperature and salinity affect gonadal development and the underlying molecular regulatory mechanisms in fish.

## 2. Results

### 2.1. Analysis of the Effect of Temperature and Salinity on Ovarian Development

**Figure 1:**
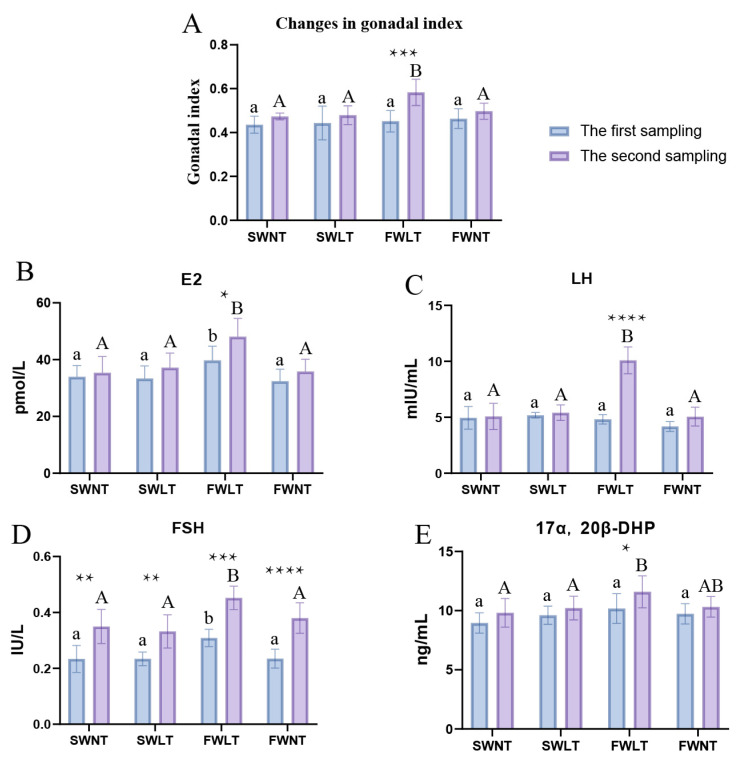
Analysis of the effects of the SWNT group (salinity: 30‰, temperature: 30 ± 1 °C), SWLT group (salinity: 30‰, temperature: 18 ± 1 °C), FWLT group (salinity: 4‰, temperature: 18), and FWNT group (salinity: 4‰, temperature: 30 ± 1 °C) on gonadosomatic indices of ovary development and hormone levels related to gonadal development of the *L. maculatus*. (**A**) Changes in gonadal indices of SWNT, SWLT, FWLT, and FWNT groups at the first sampling and at the second sampling. SWNT group, SWLT group, FWLT group, and FWNT group changes in the levels of gonadal-development-related hormones at the first sampling and at the second sampling: (**B**) E2, (**C**) LH, (**D**) FSH, and (**E**) 17α,20β-DHP. Blue color represents the first sampling, and purple color represents the second sampling. The use of lowercase letters (a, b…) indicates differences between the four treatments at the first sampling. The use of uppercase letters (A, B…) indicates the differences between the four treatments at the second sampling. The use of “*” indicates differences between the same treatments in the first and second samples (* *p* < 0.05, ** *p* < 0.01, *** *p* < 0.001, and **** *p* < 0.0001).

The gonadal indices of perch in all four groups under different salinity and temperature conditions showed an increasing trend (Figure 1A). The gonadal index of the FWLT group under brackish freshwater low-temperature conditions showed a significant increase at the end of the culture experiment (*p* < 0.001), while the three remaining groups did not significantly increase. At the first sampling, there was no significant difference (*p* > 0.05) in the gonadal indices of SWNT, SWLT, FWNT, and FWLT groups. However, at the second sampling, the gonadal indices of the FWLT group under brackish freshwater low-temperature culture conditions were significantly higher (*p* < 0.01) than those of the SWNT, SWLT, and FWNT groups under seawater normothermal, seawater low-temperature, and brackish freshwater normothermal culture conditions, respectively.

### 2.2. Effects of Temperature and Salinity on Reproduction-Related Hormones in Lateolabrax Maculatus

The levels of four hormones related to gonadal development were compared in serum samples from the first and second sampling groups of *L. maculatus*. Serum levels of the hormones E2 (Figure 1B), LH (Figure 1C), and 17α,20β-DHP (Figure 1E) showed an increasing trend in all groups, but this increase was only significant in FWLT (*p* < 0.05). Serum levels of FSH (Figure 1D) increased significantly (*p* < 0.05) in all groups. The levels of E2, LH, FSH, and 17α,20β-DHP hormones were compared in SWNT, SWLT, FWLT, and FWNT groups at the time of the second sampling. The hormone levels of E2, LH, and FSH were found to be significantly higher (*p* < 0.05) in the FWLT group than in the SWNT, SWLT, and FWNT groups, and the hormone levels of 17α,20β-DHP (Figure 1E) were significantly higher (*p* < 0.05) in the FWLT group than in the SWNT and SWLT groups.

### 2.3. Transcriptome Analysis of Ovaries at Different Temperatures and Salinities

**Figure 2:**
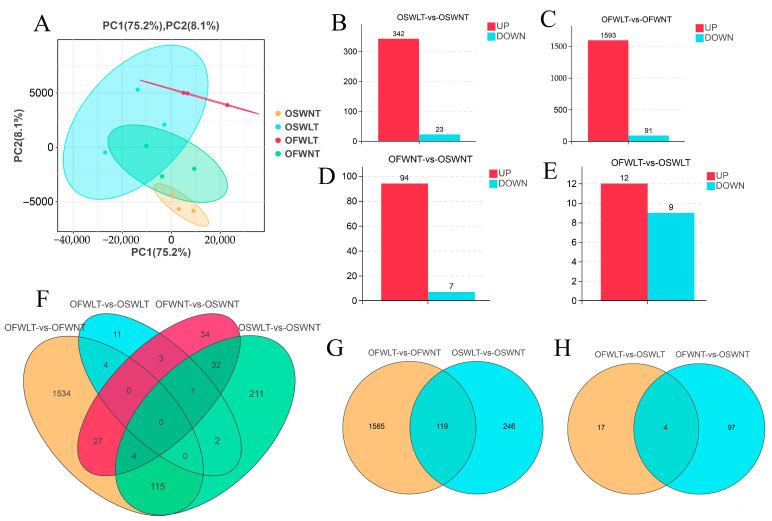
(**A**) Principal component analysis of transcriptome data for OSWNT, OSWLT, OFWLT, and OFWNT groups. Differentially expressed genes (DEGs) upregulated and downregulated in ovarian tissue of the control group under different temperature and salinity conditions: (**B**) OSWNT-vs-OSWLT, (**C**) OFWNT-vs-OFWLT, (**D**) OSWNT-vs-OFWNT, and (**E**) OSWLT-vs-OFWLT (FDR < 0.05, |log2FC| > 1). (**F**) DEGs shared between the four comparison groups. (**G**) DEGs shared by OSWNT-vs-OSWLT vs. OFWNT-vs-OFWLT. (**H**) DEGs shared by OSWNT-vs-OFWNT vs. OSWLT-vs-OFWLT. Control group is in the front, and experimental group is in the back.

To further investigate the molecular mechanisms by which temperature and salinity influence *L. maculatus* ovarian development, transcriptome analysis was performed on ovarian tissues from the SWNT, SWLT, FWNT, and FWLT groups at the final sampling point. The ovarian tissues from each group were denoted as OSWNT, OSWLT, OFWLT, and OFWNT, respectively. After quality filtering, clean reads accounted for 99.45% to 99.67% of the total raw reads. Bases with sequencing quality scores ≥ Q30 made up 92.08% to 93.32% of the total bases, and the GC content ranged from 47.56% to 51.10% (Table 1). Principal component analysis (PCA) was performed on the ovarian transcriptome data from the four groups. As shown in Figure 2A, PC1 explained 75.2% of the variance, and PC2 explained 8.1%. The OSWNT, OSWLT, OFWLT, and OFWNT groups showed high intra-group reproducibility and clear inter-group variance. These results indicate that high-quality sequence data were obtained for further analysis.

To evaluate temperature effects on ovarian development, the OSWNT and OSWLT groups (both in seawater at 30‰ salinity, but at 30 °C and 18 °C, respectively) were compared. Using the OSWNT group as the control, a total of 365 DEGs were identified in the OSWLT group, with 343 genes upregulated and 23 downregulated (Figure 2B). The OSWLT group and OFWLT group were comparison groups for the effects of different salinity levels on ovarian development under seawater conditions of 18 °C and 30‰ salinity and brackish water conditions of 4‰ salinity. Compared to the OFWNT group, 1684 DEGs were found in the OFWLT group, including 1593 upregulated and 91 downregulated genes (Figure 2C). These results revealed that the number of upregulated DEGs in the low-temperature groups was much higher than the number of downregulated DEGs under both seawater and brackish water conditions.

To examine the effects of salinity on ovarian development, the OSWNT (30‰ salinity) and OFWNT (4‰ salinity) groups were compared at 30 °C. A total of 101 DEGs were identified in the OFWNT group, with 94 genes upregulated and 7 downregulated (Figure 2D). Under low temperature (18 °C), the OSWLT and OFWLT groups were compared, and 21 DEGs were identified in the OFWLT group, with 12 genes upregulated and 9 downregulated (Figure 2E).

To further identify DEGs associated with temperature-related effects, a Venn diagram was generated using DEGs from the OSWNT vs. OSWLT and OFWNT vs. OFWLT comparisons (Figure 2G). A total of 119 DEGs were shared by both comparisons, with 246 unique to the OSWNT vs. OSWLT comparison and 1565 unique to the OFWNT vs. OFWLT comparison. Similarly, to explore DEGs related to salinity effects, a Venn diagram was constructed for the OSWNT vs. OFWNT and OSWLT vs. OFWLT comparisons (Figure 2H), identifying four DEGs common to both comparisons, 97 unique to OSWNT vs. OFWNT, and 17 unique to OSWLT vs. OFWLT.

**Figure 3:**
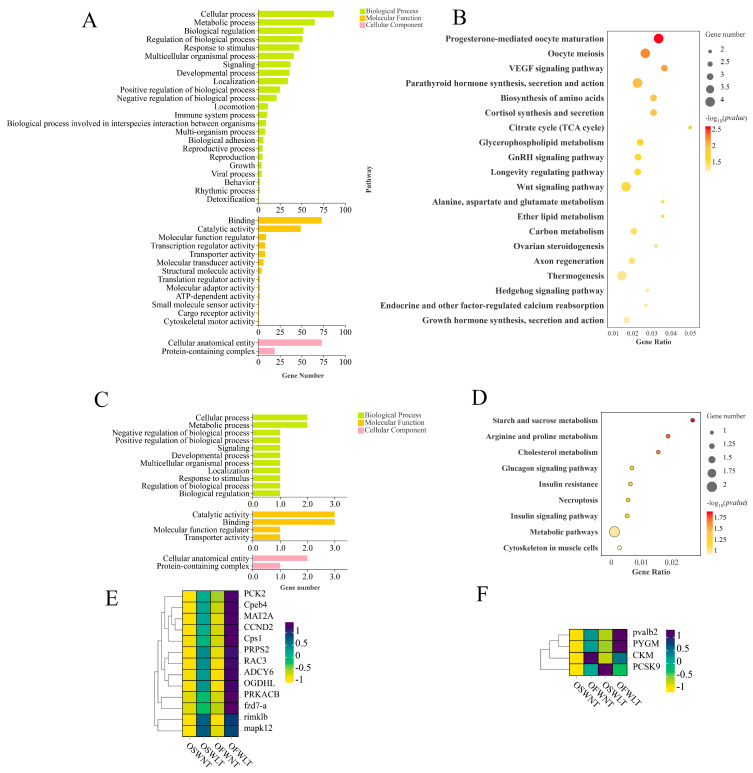
GO-enriched histograms and KEGG-enriched bubble plots of DEGs shared by OSWNT-vs-OSWLT and OFWNT-vs-OFWLT in the comparison groups of the effects of different temperatures on ovarian development. (**A**) GO enrichment histogram. (**B**) KEGG-enriched bubble plots. GO-enriched histograms and KEGG-enriched bubble plots of DEGs shared by OSWNT-vs-OFWNT and OSWLT-vs-OFWLT, comparing the effects of different salinities on gonadal development of the two groups: (**C**) GO-enriched histograms and (**D**) KEGG-enriched bubble plots. Clustering heatmap of temperature and salinity affecting DEGs in ovarian-development-related pathways: (**E**) temperature may affect DEGs in ovarian-development-related pathways and (**F**) salinity may affect DEGs in ovarian-development-related pathways.

GO enrichment analysis was performed on DEGs common to the two groups comparing temperature effects on gonadal development (Figure 3A). Biological processes were primarily enriched in cellular processes, metabolic processes, and biological regulation. Molecular functions were enriched in binding, catalytic activity, and regulatory activities, while cellular components were enriched in anatomical structures and protein-containing complexes. KEGG enrichment analysis of these temperature-responsive DEGs revealed significant enrichment in energy metabolism pathways (the citric acid cycle and central carbon metabolism), amino acid biosynthesis, and cell proliferation/differentiation pathways (Wnt and Hedgehog signaling). Additionally, ovarian-development-related pathways—including progesterone-mediated oocyte maturation, ovarian steroidogenesis, and oocyte meiosis—were enriched (Figure 3B).

GO enrichment analysis of DEGs common to the two salinity comparison groups showed enrichment in cellular metabolism and regulation, binding, catalytic activity, and protein-containing complexes (Figure 3C). KEGG analysis indicated significant enrichment in arginine and proline metabolism, cholesterol metabolism, and the insulin signaling pathway (Figure 3D).

DEGs from temperature- and salinity-responsive pathways were visualized in clustered heatmaps. The heatmap of temperature-responsive pathways (Figure 3E) showed that 12 DEGs were upregulated in the low-temperature groups (OSWLT and OFWLT), with the highest expression in OFWLT, and downregulated in the normothermic groups (OSWNT and OFWNT). The salinity-related heatmap (Figure 3F) revealed four DEGs upregulated in the brackish-water groups (OFWNT and OFWLT) and downregulated in seawater groups (OSWNT and OSWLT). Notably, *PCSK9* expression exhibited opposite patterns between conditions: it was downregulated in OSWNT, upregulated in OFWNT, upregulated in OSWLT, and downregulated in OFWLT.

**Figure 4:**
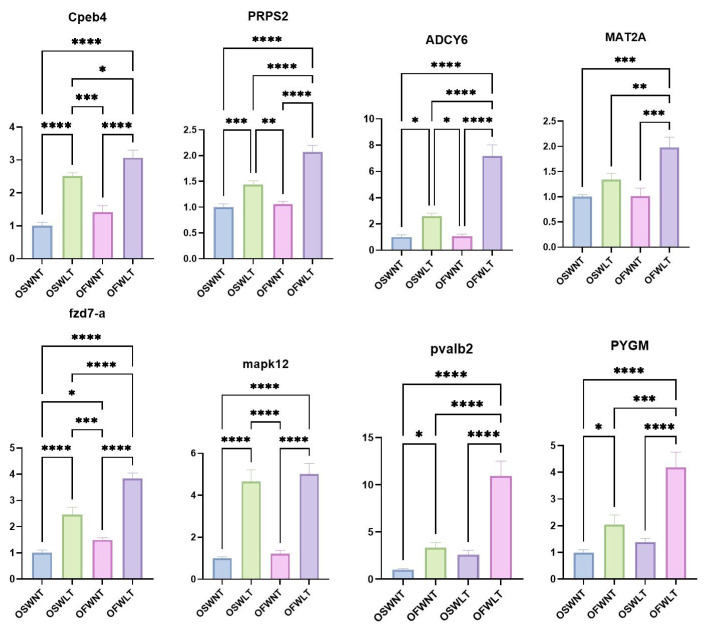
Validation of RT-qPCR analysis of DEGs in ovarian transcriptome (* *p* < 0.05, ** *p* < 0.01, *** *p* < 0.001, and **** *p* < 0.0001).

Eight DEGs (*Cpeb4*, *PRPS2*, *ADCY6*, *MAT2A*, *FZD7-A*, *MAPK12*, *PVALB2*, and *PYGM*) were selected from the *L. maculatus* ovarian transcriptome. β-Actin served as the internal reference gene, and primer sequences are provided in Table 2. The transcriptome data were validated by RT-qPCR. Clustering heatmaps (Figure 3E,F) and RT-qPCR results (Figure 4) confirmed that the expression patterns of these eight genes were consistent with the RNA-seq data.

### 2.4. Transcriptome and Energy Metabolism Analysis of Ovarian and Muscle Tissues During Ovarian Development

**Figure 5:**
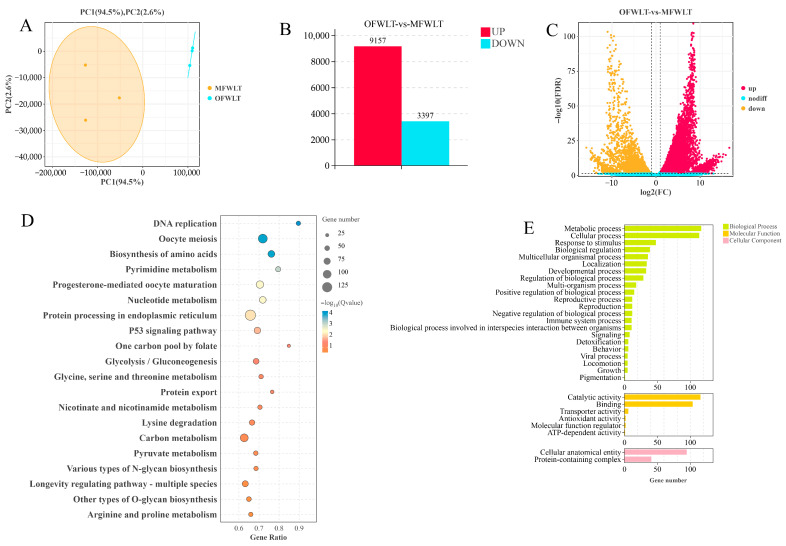
(**A**) Principal component analysis of the transcriptome of OFWLT and MFWLT. Differentially expressed genes (DEGs) between upregulation and downregulation in OFWLT and MFWLT: (**B**) Bar graph of DEGs and (**C**) DEGs volcano plot. GO enrichment analysis and KEGG enrichment analysis of DEGs in OFWLT and MFWLT: (**D**) GO enrichment histogram and (**E**) KEGG bubble diagram.

To investigate the mechanisms of energy metabolism between ovaries and muscles during ovarian development, transcriptome analysis was performed on muscle and ovary tissues collected at the second sampling from the FWLT group of *L. maculatus*. OFWLT represented ovarian tissue, and MFWLT represented muscle tissue from the FWLT group. After data filtering, clean reads accounted for 98.59–99.09% of the raw transcriptome sequencing reads. The bases with sequencing quality values of Q30 or above accounted for 92.22–93.09% of the total bases. The GC content ranged from 47.56% to 52.27% of the total bases, indicating high data quality suitable for subsequent analysis (Table 3). PCA was performed on the transcriptomic data of OFWLT and MFWLT, as shown in Figure 5A. PC1 explained 94.5% of the variance, capturing most of the variation in the data. PC2 explained 2.6% of the variance, capturing additional variation. The OFWLT and MFWLT groups showed good intra-group sample reproducibility and significant inter-group differences. These results indicate that high-quality sequence data were obtained for further analysis.

Using MFWLT as the control group, a total of 12,554 differentially expressed genes (DEGs; FDR < 0.05 and |log2FC| > 1) were identified from the transcriptome comparison between ovary and muscle tissues in the FWLT group. Among these, 9157 genes were upregulated and 3397 genes were downregulated, with the number of upregulated genes significantly exceeding that of downregulated genes (Figure 5B). GO enrichment analysis of the DEGs (Figure 5E) revealed that biological processes were mainly enriched in cellular and metabolic processes. Molecular functions were predominantly enriched in binding and catalytic activities, while cellular components were mainly enriched in cellular anatomical entities and protein-containing complexes. KEGG enrichment analysis of the DEGs (Figure 5D) showed significant enrichment in energy-metabolism-related pathways, including glycolysis/gluconeogenesis, carbon metabolism, and pyruvate metabolism.

**Figure 6:**
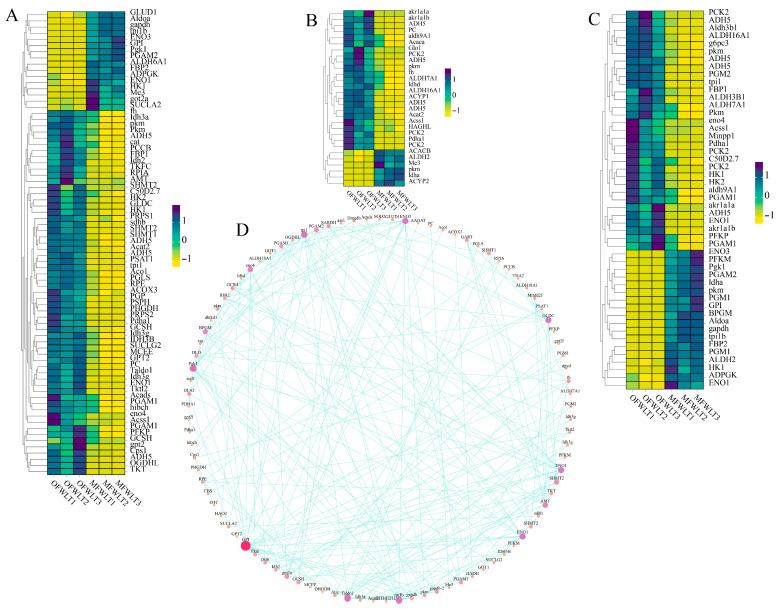
Heatmap of energy-metabolism-related pathways up- and down-regulating DEGs: (**A**) carbon metabolism, (**B**) pyruvate metabolism, and (**C**) glycolysis/glycolysis. (**D**) PPI network interactions graph of DEGs in glycolysis/glycolysis, carbon metabolism, and pyruvate metabolism pathways. Node names represent gene names, node size and color shades represent gene connectivity, and line segments represent gene–gene interactions. The greater the gene connectivity, the larger and darker the node size and color.

DEGs significantly enriched in the carbon metabolism (Figure 6A), pyruvate metabolism (Figure 6B), and glycolysis/gluconeogenesis (Figure 6C) pathways were compiled into separate gene sets. Heatmaps illustrating differential gene expression for each pathway were then generated. In total, 75 DEGs were identified in the carbon metabolism pathway, with 59 upregulated and 16 downregulated genes. The glycolysis/gluconeogenesis pathway contained 49 DEGs, including 31 upregulated and 18 downregulated genes. In the pyruvate metabolism pathway, 29 DEGs were found, of which 23 were upregulated and 6 downregulated.

DEGs from the glycolysis/gluconeogenesis, carbon metabolism, and pyruvate metabolism pathways were combined into a new gene set for protein–protein interaction (PPI) analysis with the overall DEGs. The top 200 genes ranked by combined score were selected to construct the PPI network interaction map (Figure 6D). Sixteen potential genes regulating energy metabolism with gene connectivity greater than six were identified from the PPI interaction map: *GPI*, *Got2a*, *GCSH*, *Taldo1*, *Tpi1b*, *PGAM1*, *ENO1*, *AMT*, *SHMT2*, *GLDC*, *ENO3*, *PGAM2*, *Tpi1*, *ENO4*, *BPGM*, and *Pgk1*.

**Figure 7:**
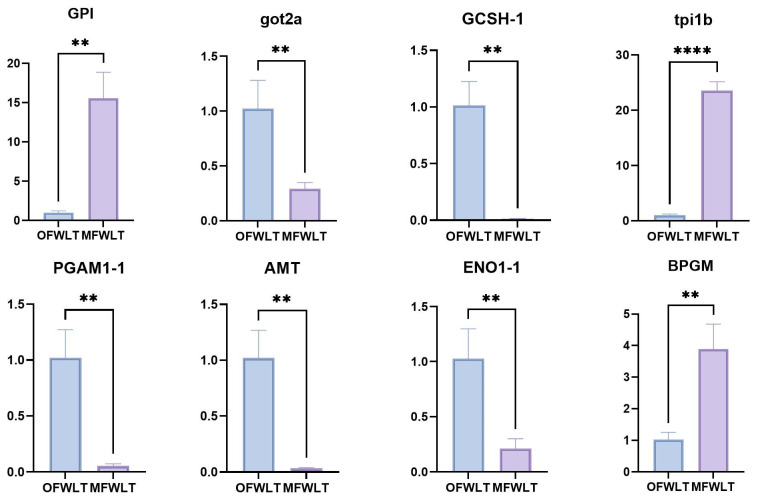
Validation of RT-qPCR analysis of DEGs expressed genes in the OFWLT and MFWLT groups (** *p* < 0.01 and **** *p* < 0.0001).

Eight genes were selected from the energy-metabolism-related pathway DEGs for RT-qPCR analysis to verify the reliability of the transcriptome results (Figure 7). β-Actin was used as the internal reference gene, with primer sequences listed in Table 3. Combined with the heatmap of energy-metabolism-related pathway DEG expression (Figure 6A–C), analysis showed that the expression trends of the eight genes in RT-qPCR were generally consistent with the transcriptome analysis results, indicating that the RNA-seq data are reliable.

**Figure 8:**
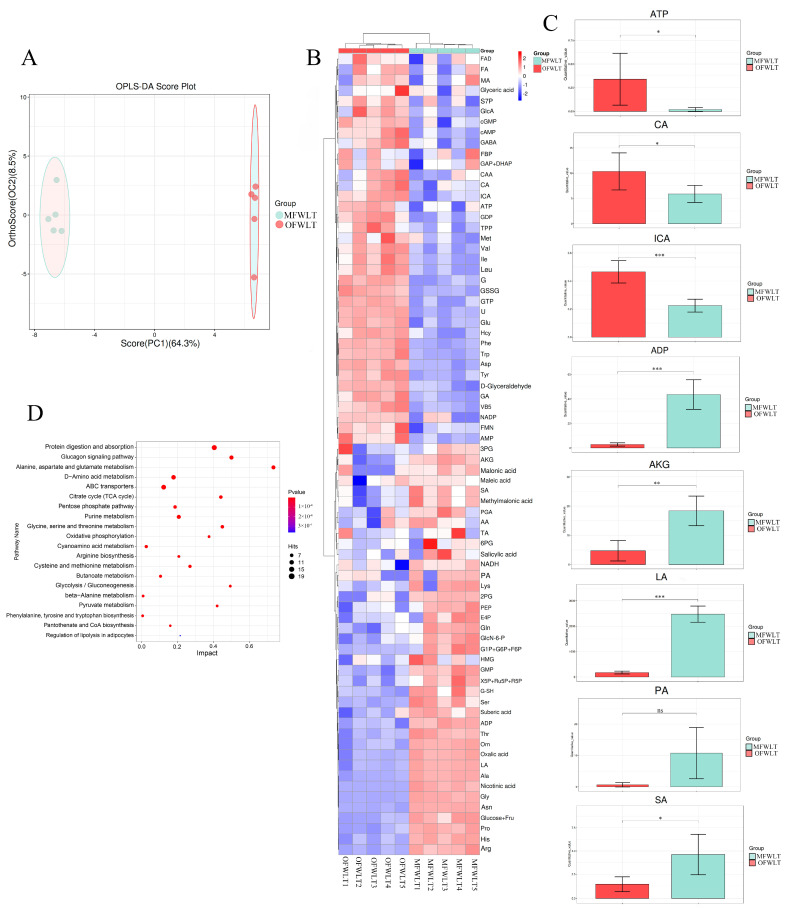
(**A**) Analysis of OFWLT and MFWLT OPLS-DA. (**B**) Heatmap of different metabolite clustering between OFWLT and MFWLT groups. (**C**) OFWLT vs. MFWLT metabolite level box plot. (**D**) KEGG-enriched bubble plot of OFWLT vs. MFWLT group differential metabolites.

OPLS-DA of the targeted energy metabolomics data from ovaries and muscles of the FWLT group (Figure 8A) showed that the first principal component explained 64.3% of the total variance, with a Q2 value of 0.99, demonstrating the model’s reliability. Samples clustered closely within each group, indicating good intra-group reproducibility. The ovary and muscle samples were clearly separated, showing distinct grouping and confirming the reproducibility of the targeted energy metabolism data.

The detected energy metabolites were plotted as differential metabolite heatmaps (Figure 8B). A total of 76 metabolites related to energy metabolism were detected in the analysis. Compared to muscle tissue, 37 metabolites were upregulated and 39 were downregulated in the ovaries of the FWLT group, indicating significant differences in energy metabolism between the two tissues.

ATP content in the ovaries of the FWLT group (Figure 8C) was significantly higher than in muscle (*p* < 0.05), while ADP content was significantly lower (*p* < 0.001). Citric acid (CA) and isocitrate (ICA) contents were significantly higher in the ovary than in muscle (*p* < 0.05), whereas α-ketoglutarate (AKG), succinic acid (SA), and lactic acid (LA) contents were significantly lower (*p* < 0.05). Pyruvic acid (PA) content was lower in the ovary but not significantly different from muscle (*p* > 0.05).

KEGG enrichment analysis (Figure 8D) revealed significant enrichment in pathways, including the TCA cycle, pentose phosphate pathway, oxidative phosphorylation, glycolysis/gluconeogenesis, amino acid metabolism, fatty acid oxidation (FAO), and other major cellular energy acquisition pathways.

### 2.5. Combined Transcriptome and Energy Metabolome Analysis

**Figure 9:**
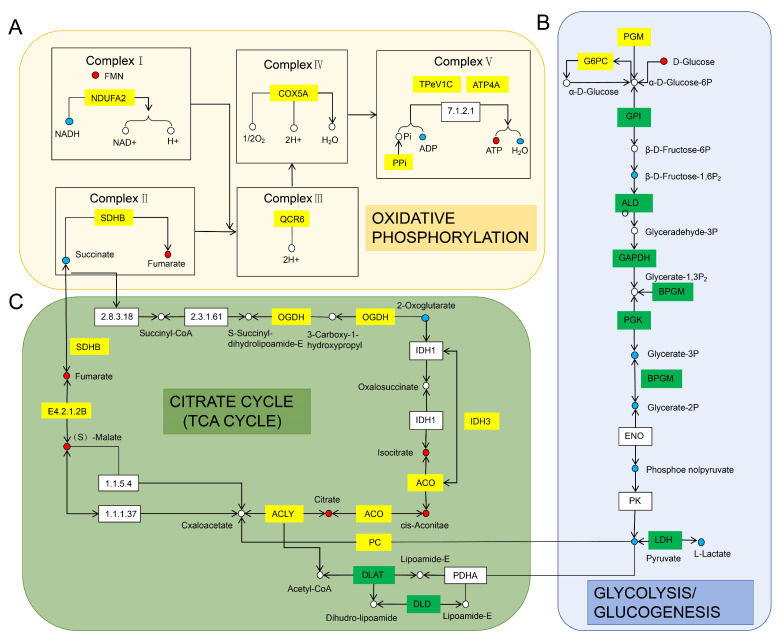
Pathway maps for OFWLT and MFWLT transcriptome and target energy metabolome association analysis. (**A**) Oxidative phosphorylation, (**B**) glycolysis/gluconeogenesis, and (**C**) TCA cycle. Red circles indicated upregulated metabolites, blue circles indicated downregulated metabolites, and white circles represented metabolites not detected in this study. Yellow squares represented upregulated genes, green squares indicated downregulated genes, and white squares represented genes with no or min-imal changes.

Genes upregulated in the oxidative phosphorylation pathway (Figure 9A) included *NDUFA2*, *SDHB*, *QCR6*, *PPA*, *TPEV1C*, and *ATP4A*. Metabolites upregulated in this pathway included flavin mononucleotide (FMN), fumarate, and ATP, while downregulated metabolites included NADH, SA, ADP, and H_2_O.

Genes upregulated in the TCA cycle pathway (Figure 9B) included *PC*, *ACO*, *IDH3*, *ACLY*, *SDHB*, and *E4.2.1.2B*, whereas downregulated genes included *DLAT* and *DLD*. Metabolites upregulated in the TCA cycle included isocitrate (ICA), cis-aconitic acid (CAA), citric acid (CA), malic acid (MA), and fumaric acid (FA), whereas downregulated metabolites included succinic acid (SA), pyruvic acid (PA), and α-ketoglutarate (AKG).

In the glycolysis/gluconeogenesis pathway (Figure 9C), upregulated genes included *PGM2* and *G6PC*. Downregulated genes included *GIP*, *ALDO*, *GAPDH*, *BPGM*, *PGK*, *LDH*, *DLAT*, and DLD. Upregulated metabolites in this pathway included D-glucose, while downregulated metabolites included beta-D-fructose 1,6-bisphosphate (BDFP), 3-phospho-D-glycerate, 2-phospho-D-glycerate, pyruvate, and L-lactate.

## 3. Discussion

### 3.1. Analysis of the Effects of Temperature and Salinity on Ovarian Development

In this study, we investigated the effects of environmental factors on the ovarian development of *L. maculatus* by exposing them to different salinity and temperature conditions. The results showed that although the gonadosomatic index (GSI) tended to increase in all four experimental groups, a significant increase was observed only in the FWLT group (salinity: 4‰, temperature: 18 ± 1 °C). This indicates that a single environmental factor—either low temperature or low salinity alone—is not sufficient to significantly promote ovarian development.

In contrast, the gonadal indices of the FWLT group (salinity: 4‰, temperature: 18 ± 1 °C) were significantly higher than those of the other groups, suggesting that temperature and salinity may synergistically affect the ovarian development of *L. maculatus*. Some studies have reported that there may be an interaction between salinity and temperature to jointly promote ovarian development [21]. Temperature has been shown to be a key environmental factor influencing the reproductive cycle of fish, and changes in unsuitable temperatures can affect the reproductive development of fish through the endocrine system, especially by suppressing the production of the ovarian estrogen [5]. At the same time, Varsamos et al. suggested that low-salinity environments may reduce osmoregulatory energy consumption in fish, thus allowing more energy to be used for reproductive development [36]. *O. mykiss*, on the other hand, use less energy for respiration at low salinity than at high salinity, consume significantly more energy for growth, and have higher protein, lipid, and energy content in their bodies [37]. Female *Lateolabrax japonicus* also showed enhanced oocyte maturation and spawning at lower salinity [38]. The above findings may explain the significant enhancement of ovarian development in the FWLT group under low-salinity and low-temperature conditions.

Several studies have shown that FSH plays a major regulatory role in the early stages of gonadal development and gametogenesis in fish, while LH is mainly involved in the final stages of gamete maturation. In *S. salar*, FSH serum levels increase with ovarian development, whereas plasma LH is undetectable or very low during early gametogenesis and rises significantly during late gametogenesis [39]. In female *S. salar*, only FSH is present in the blood of sexually immature fish. Plasma levels of FSH increase during the yolk formation stage and decrease during the follicle maturation and spawning stages [40,41]. Serum E2 levels also increased during ovarian development in female *L. maculatus* [13]. The 17α,20β-DHP increased gradually during gametogenesis in *O. mykiss*, reaching a maximum at maturity and declining after spawning [42]. The active ovarian development in the FWLT group was further verified by serum hormone testing, with E2, LH, and 17α,20β-DHP all significantly elevated in this group (*p* < 0.05), whereas no significant changes were observed in the other groups. E2 is a key hormone for yolk protein synthesis and follicular development, LH is closely related to final follicular maturation and ovulation, and 17α,20β-DHP is considered a maturation-inducing hormone in fish. The elevation of these hormones in the FWLT group indicated significant activation of its reproductive axis (HPG axis), supporting the observed increase in GSI. In addition, FSH was significantly elevated (*p* < 0.05) in all experimental groups, suggesting that *L. maculatus* entered the initial follicular development stage under different environmental conditions. However, synchronized elevation of other hormone levels was observed only in the FWLT group, suggesting that low temperature and low salinity are important conditions promoting the transition from early development to maturity of the *L. maculatus* ovary.

### 3.2. Analysis of the Effects of Temperature and Salinity on the Ovarian Transcriptome

#### 3.2.1. Analysis of the Effect of Temperature on the Ovary Transcriptome

To further investigate the effect of temperature on ovarian development, differentially expressed genes (DEGs) common to both temperature comparison groups were selected to construct a core gene set. A total of 119 DEGs were identified simultaneously in both the SWNT vs. SWLT and FWNT vs. FWLT comparison groups, suggesting that these genes may be core regulators of the ovarian response to temperature changes in *L. maculatus*. KEGG analysis showed significant enrichment of pathways related to energy metabolism (citric acid cycle and carbon metabolism), amino acid biosynthesis, and cell proliferation and differentiation pathways (Wnt and Hedgehog). Additionally, pathways closely related to ovarian development, such as the progesterone-mediated oocyte maturation pathway, ovarian steroidogenesis, and oocyte meiosis pathway, were significantly enriched.

The ovarian steroidogenesis pathway is involved in the biosynthesis of steroid hormones, such as estrogen and progesterone, in the ovary [43]. Examination of sex hormone levels in both low- and high-temperature groups revealed a significant increase in hormone levels in the FWLT group, suggesting that low temperature may promote the synthesis and secretion of the sex steroid hormones by modulating the ovarian steroidogenesis pathway. In fish, progesterone and its derivatives are the most critical signaling steroids initiating final oocyte maturation (FOM) and ovulation. It has been suggested that external environmental factors may also regulate the maturation process of fish oocytes by influencing the progesterone signaling pathway [44,45], and temperature may be one of these exogenous factors.

ADCY6 is a membrane-bound adenylate cyclase that catalyzes the conversion of ATP to cyclic adenosine monophosphate (cAMP), a key second messenger in hormone signaling pathways, such as gonadotropin-releasing hormone (GnRH), FSH, and LH [46]. In fish ovaries, cAMP promotes the expression of steroidogenic enzymes, thereby enhancing the synthesis of sex hormones, such as E2 and progesterone (P) [47]. The high expression of *ADCY6* in the low-temperature groups suggests that the cAMP pathway may be activated, providing a signaling foundation for steroidogenesis. cAMP initiates downstream phosphorylation by activating PRKACB, a catalytic isoform of protein kinase A (PKA) [48]. PKA activates several transcription factors, which enhance the transcriptional activity of the *steroidogenic acute regulatory* (*StAR*) gene and promote steroid hormone synthesis [49]. The upregulation of *ADCY6* and *PRKACB* at 18 °C suggests that the cAMP-PKA signaling pathway may be more active in the ovaries of *L. maculatus* at this temperature, thereby enhancing steroid hormone synthesis. This finding is consistent with the observed significant elevations of E2 and 17α,20β-DHP at 18 °C. It reflects that the regulatory effect of temperature on steroid synthesis may be mediated through the ADCY6-cAMP-PRKACB pathway.

Meiotic progression is driven by the sequential translational activation of maternal messenger RNAs (mRNAs) stored in the cytoplasm. This activation is primarily induced by cytoplasmic elongation of the poly(A) tail, which is mediated by the cytoplasmic polyadenylation element (CPE) located in the 3′ untranslated region. Cytoplasmic polyadenylation element-binding protein 4 (Cpeb4), a member of the CPEB family of proteins, mainly regulates polyadenylation and translation of mRNAs. It has been shown that during meiosis, the expression of *CPEB1* gradually decreases while the expression of *CPEB4* gradually increases, thus taking over CPEB1’s function. *CPEB4* regulates the translation of mRNAs during later meiotic stages, ensuring oocyte maturation [50]. At low-temperature conditions, the upregulation of Cpeb4 may enhance the translational control of specific mRNAs, thus affecting the proliferation and differentiation of ovarian cells to adapt to the developmental needs of the ovary.

Mitogen-activated protein kinase 12 (MAPK12) belongs to the p38 MAPK family, involved in the cellular stress response and cell cycle regulation. MAPK12 plays a key role in the G2-to-M phase transition in oocytes by regulating phosphorylation of related proteins. The MAPK pathway is essential for the G2-to-M phase transition in oocytes, regulating phosphorylation of related proteins and promoting meiosis [51]. The specific function of MAPK12 in oocyte meiosis has not been fully investigated. However, MAPK-activated protein kinase 2 (MAPK2) has been found essential for meiotic bipolar spindle formation, chromosome segregation, and proper mitotic–microtubule attachment during oocyte meiosis in Mus musculus [52]. MAPK12 and MAPK2 are both members of the p38 MAPK family. MAPK2 is in the same p38 MAPK family, and given the MAPK pathway’s role in oocyte meiosis, MAPK12 may also regulate oocyte meiosis, potentially mediating temperature effects. The specific function and mechanisms of MAPK12 in oocyte meiosis warrant further investigation via gene knockdown and overexpression studies.

The amino acid biosynthesis pathway provides amino acids necessary for protein synthesis, supporting cell proliferation, differentiation, and function [53]. Phosphoribosyl pyrophosphate synthase 2 (PRPS2) is a key rate-limiting enzyme in nucleotide biosynthesis, catalyzing the reaction of ribulose-5-phosphate with ATP to form phosphoribosyl pyrophosphate (PRPP). PRPP is not only a precursor for purine and pyrimidine nucleotide synthesis but is also involved in the biosynthesis of certain amino acids (histidine and tryptophan) [54]. The high expression of PRPS2 at low temperature indicates active oocyte proliferation and nucleic acid synthesis to support rapid ovarian tissue development. Although no direct studies report the role of PRPS2 in fish ovarian development, its high expression in other rapidly proliferating tissues supports its potential importance in ovarian development [55]. Methionine adenosyltransferase 2A (MAT2A) encodes S-adenosylmethionine (SAM) synthetase, a key enzyme in one-carbon metabolism and the methionine cycle. SAM is a major methyl donor in cells and is widely involved in methylation of DNA, RNA, and histones, regulating gene expression and cell differentiation [56]. Upregulation of MAT2A enhances methyl donor synthesis, supporting epigenetic regulation and metabolic homeostasis. This may be important for ovarian function and oocyte maturation, though its specific role requires further study.

Carbamoyl phosphate synthetase 1 (CPS1) is a mitochondrial enzyme and a key component of the urea cycle, catalyzing the production of carbamoyl phosphate from ammonia and bicarbonate, which enters ammonia excretion and nitrogen metabolism pathways [57]. Protein synthesis and amino acid metabolism are significantly enhanced during ovarian development. The high expression of CPS1 at 18 °C may help remove ammonia produced during metabolism, maintain intracellular nitrogen balance, and optimize the ovarian microenvironment. Although direct studies on CPS1 in fish ovarian development are lacking, its critical role in nitrogen metabolism supports its potential importance in ovarian development.

The tricarboxylic acid (TCA) cycle is a fundamental metabolic pathway that plays a central role in cellular respiration [58], and the ATP it produces is the primary energy currency of the cell [59]. Beyond energy production, the TCA cycle also supports a wide range of biosynthetic processes by providing precursors for amino acids, nucleotide bases, and other essential biomolecules [60]. Oxoglutarate dehydrogenase-like (OGDHL), a mitochondrially localized enzyme, functions analogously to oxoglutarate dehydrogenase (OGDH) within the alpha-ketoglutarate dehydrogenase complex, exhibiting similar catalytic activity [61]. It catalyzes the conversion of AKG to succinyl-CoA with the concomitant production of NADH, representing one of the rate-limiting steps in the TCA cycle [62]. Oocytes and peripheral supporting cells require large amounts of ATP for processes such as RNA/DNA synthesis, protein translation, and vesicular transport during growth and differentiation. Upregulation of *OGDHL* expression may indicate enhanced TCA cycle activity, potentially accelerating oxidative phosphorylation via increased NADH production and thereby supplying sufficient energy and intermediate metabolites for cell proliferation, hormone synthesis, and meiosis. Currently, studies investigating the expression and functional roles of *OGDHL* in ovarian tissues are lacking. Its potential involvement in ovarian development and oocyte maturation, particularly in energy metabolism and redox homeostasis, warrants further investigation. Phosphoenolpyruvate carboxykinase 2 (PCK2) catalyzes the conversion of oxaloacetate (OAA), a TCA cycle intermediate, into phosphoenolpyruvate (PEP), thereby linking gluconeogenesis to the TCA cycle [63,64]. Upregulation of *PCK2* in ovarian tissues at 18 °C may facilitate the diversion of accumulated OAA into PEP, thereby supporting the pentose phosphate pathway (PPP), which provides ribose-5-phosphate for nucleotide synthesis and NADPH for biosynthesis of amino acids, fatty acids, and cholesterol [65].

The Wnt and Hedgehog (Hh) signaling pathways are classical regulatory mechanisms involved in ovarian follicle development. Both pathways interact with FOXO3, a transcription factor regulated by the PI3K/AKT pathway, which is considered a central regulator of ovarian dormancy and primordial follicle activation [66]. Several components of the Wnt pathway have been reported to be upregulated in *O. mykiss* ovaries during follicular development, indicating its critical involvement in ovarian function [67]. WNT proteins are secreted glycoproteins that regulate multiple signaling cascades via β-catenin-dependent, β-catenin-independent, and WNT/Ca^2+^-mediated mechanisms [68]. The canonical WNT/β-catenin signaling pathway is an evolutionarily conserved system of intercellular communication that plays essential roles in regulating cell proliferation, differentiation, stem cell renewal, motility, and apoptosis during embryogenesis and adult tissue homeostasis [69]. Activation of protein kinase A (PKA) has been shown to inhibit β-catenin ubiquitination, resulting in its intracellular accumulation [70]. The Hh signaling pathway is also evolutionarily conserved and regulates embryonic development as well as fundamental cellular processes, such as proliferation, differentiation, and survival. Disruption of Hh signaling is associated with severe developmental abnormalities and disease pathogenesis [71]. Effective communication among oocytes, granulosa cells, and theca cells is essential for proper follicular development. Hh signaling contributes significantly to this intercellular interaction, particularly between granulosa and theca cells [72]. Fzd7-a belongs to the Frizzled receptor family and is a seven-pass transmembrane protein. It binds Wnt ligands and activates the canonical β-catenin-dependent Wnt signaling pathway via the co-receptor LRP5/6 [73]. Upon activation, β-catenin is stabilized in the cytoplasm and translocates into the nucleus, promoting transcription of target genes such as *cyclin D1* (*CCND1*) and *Myc*, which are essential for cell proliferation, differentiation, and migration. In addition to the canonical pathway, Fzd7-a also participates in non-canonical Wnt signaling, including the Wnt/PCP and Wnt/Ca^2+^ pathways, regulating cell polarity, motility, and morphological remodeling [74]. RAC3, a small Rho family GTPase, plays critical roles in cytoskeletal reorganization during oocyte maturation [75] and contributes to EGF receptor activation [76]. In fish, EGF regulates oocyte final maturation (OFM) and ovulation [77,78]. In the non-canonical Wnt/PCP pathway, ligand binding to FZD and ROR/Ryk co-receptors facilitates the assembly of PCP complexes, including Vangl, Prickle, Celsr, and DVL [73]. Dishevelled (DVL), a downstream cytoplasmic protein, interacts with RAC to coordinate cell polarity and motility [79]. In our study, *PRKACB*, *RAC3*, and *fzd7-a* were significantly upregulated in the low-temperature group. *PRKACB* may enhance PKA activity, promoting β-catenin stabilization. Meanwhile, *Fzd7-a* likely activates both canonical and non-canonical Wnt pathways, contributing to oocyte proliferation, migration, and morphogenesis, in part by upregulating *RAC3*. CCND2, a D-type cyclin, regulates G1/S phase transition and is essential for granulosa cell proliferation during folliculogenesis [80]. In mice, its expression is induced by FSH via a cAMP-dependent mechanism [81]. The Hh pathway, through ligands such as Shh and Ihh, activates Gli1/Gli2 transcription factors, which have been shown to promote *CCND2* expression [82]. Thus, upregulation of *CCND2* in the low-temperature group may reflect enhanced follicular cell proliferation and differentiation, potentially via FSH and Hh signaling.

#### 3.2.2. Effect of Salinity on Ovarian Transcriptome

To further explore the impact of salinity on ovarian development, a gene set was constructed using differentially expressed genes (DEGs) shared across both temperature comparison groups. Among the two salinity comparison groups, only four DEGs showed significant changes in both the OSWNT vs. OFWNT and OSWLT vs. OFWLT comparisons, suggesting that these genes may serve as core regulators in the ovarian response to salinity changes. KEGG pathway enrichment analysis revealed that these DEGs were significantly associated with arginine and proline metabolism, cholesterol metabolism, and the insulin signaling pathway—all of which are functionally relevant to ovarian development and hormone synthesis in fish.

Amino acids (AAs) serve not only as protein building blocks, but also as essential precursors for the synthesis of biologically active compounds, such as cytokines and antibodies, playing key roles in physiological regulation [83]. Arginine, in particular, is a precursor for creatine, polyamines, and nitric oxide (NO), and contributes to the creatine–phosphocreatine energy system [84]. Arginine is converted to NO via nitric oxide synthase (NOS), which stimulates the secretion of GnRH and LHRH, thereby modulating pituitary gonadotropin release [85]. In *Artemia sinica*, genes involved in the arginine biosynthesis pathway were upregulated under low-salinity conditions and downregulated under high salinity [21], a trend also observed in *Exopalaemon carinicauda* [86], indicating a role for arginine metabolism in the ovarian response to salinity. CKM (creatine kinase, muscle type) catalyzes the reversible phosphorylation of creatine and ATP to form phosphocreatine (PCr) and ADP, constituting an intracellular energy buffer system [87]. PCr functions as a reservoir of high-energy phosphates, providing energy to tissues with high ATP demand [88]. In Chinese sturgeon, increased arginine metabolism during ovarian development correlated with elevated levels of creatine-related metabolites and the expression of creatine synthase (*GATM*), underscoring the importance of the creatine–phosphocreatine system in ovarian energy supply [89]. As energy demand increases during oocyte maturation and steroid hormone synthesis, upregulation of *CKM* in the low-salinity group may enhance ATP replenishment through improved phosphocreatine cycling. Furthermore, enhanced arginine metabolism may elevate creatine levels, thereby promoting PCr synthesis and supporting ovarian bioenergetics.

PCSK9 is a protease involved in the regulation of cholesterol metabolism and indirectly increases plasma cholesterol levels by promoting the degradation of low-density lipoprotein receptors (LDLR) [90]. Several studies have demonstrated a positive correlation between PCSK9 expression and serum cholesterol levels [91]. Cholesterol serves as a precursor for sex steroid biosynthesis, with the process initiated by the rate-limiting transport of cholesterol into mitochondria, a step mediated by StAR. Within mitochondria, cholesterol is converted to pregnenolone, the first intermediate in the steroidogenic cascade and a major rate-limiting step in sex steroid formation [92]. CYP11A1, the cholesterol side-chain cleavage enzyme, catalyzes this conversion and represents the only enzyme responsible for transforming cholesterol into pregnenolone. Due to the low efficiency of this enzymatic step, CYP11A1 plays a critical role in determining the overall rate of steroid hormone synthesis [93]. Based on these biochemical mechanisms, upregulation of PCSK9—by increasing plasma cholesterol—may favor sex steroid production. In our study, PCSK9 expression exhibited distinct patterns under different environmental conditions. At 30 °C, PCSK9 was significantly upregulated in the low-salinity FWNT group. In contrast, at 18 °C, its expression was higher in the high-salinity SWLT group and downregulated in the low-salinity FWLT group relative to SWLT. Although increased PCSK9 expression in the SWLT group should theoretically enhance sex steroid production, our results revealed higher sex steroid levels in the FWLT group, suggesting that other regulatory mechanisms may be involved. This inconsistency may reflect a synergistic effect of temperature and salinity on sex steroid synthesis. Our findings suggest that low temperature acts as a primary factor driving *PCSK9* upregulation in the ovary, while high salinity also promotes *PCSK9* expression but only in the context of low-temperature conditions. Although the molecular mechanisms through which temperature and salinity regulate *PCSK9* expression remain to be elucidated, our results underscore the importance of further research into how environmental factors influence *PCSK9*-mediated regulation of ovarian function in fish.

Insulin was initially recognized for its role in maintaining glucose, lipid, and energy homeostasis in tissues, such as muscle, liver, and adipose tissue [94]. In the ovary, insulin exerts pro-steroidogenic effects mediated through MAPK and AKT signaling pathways [95]. During later stages of oocyte development, insulin promotes oocyte growth by increasing the number of hormone receptors or enhancing the sensitivity and binding affinity of luteinizing hormone (LH) receptors [96]. It also acts synergistically with follicle-stimulating hormone (FSH) to promote the differentiation and proliferation of ovarian mesenchymal stromal cells [97]. Additionally, insulin stimulates both steroidogenesis and cell proliferation in cultured granulosa and theca cells [94]. Elevated insulin levels are associated with reduced follicular atresia, thereby increasing the overall number of viable follicles [98,99]. Muscle glycogen phosphorylase (PYGM) is a key enzyme in glycogen metabolism, catalyzing the breakdown of glycogen into glucose-1-phosphate (G1P), which enters glycolysis or gluconeogenesis to supply energy [100]. PYGM activity is indirectly regulated by insulin signaling, which suppresses glycogenolysis via activation of protein phosphatase 1 (PP1), leading to PYGM dephosphorylation and enzymatic inhibition [101]. The observed upregulation of *PYGM* under low-salinity conditions may indicate a reduction in insulin-mediated suppression, possibly reflecting the increased energy demands of ovarian tissue under low temperature or specific salinity stress. This metabolic shift may facilitate energy production necessary for oocyte maturation and steroid hormone synthesis.

Pvalb2, a member of the β-type parvalbumin family, functions as a high-affinity calcium-binding protein. In *Lates calcarifer*, pvalb2 expression is normally restricted to muscle, brain, and intestine [102]. However, our transcriptomic and qPCR analyses demonstrated pvalb2 expression in the ovarian tissue of this species, with significantly higher levels under low salinity. Although limited, current evidence suggests that pvalb2 reduces intracellular Ca^2+^ levels, thereby facilitating muscle relaxation, and is more abundant in fast-relaxing muscles [103,104]. Given its calcium-binding properties and differential expression, we hypothesize that pvalb2 may also play a role in osmoregulation within germ cells. Nevertheless, its exact function in ovarian physiology remains to be determined.

### 3.3. Transcriptome and Energy Metabolome Analysis of Gonadal and Muscle Tissues During Gonadal Development

#### 3.3.1. Transcriptome Analysis of Gonadal and Muscle Tissue

Energy metabolism is fundamental to sustaining all biological activities in organisms [105]. Among the four groups of *L. maculatus* exposed to varying temperature and salinity conditions, the gonads in the FWLT group exhibited the most advanced development. As such, this group was selected to explore the mechanisms of energy metabolism during the trade-off between growth and reproduction.

Transcriptomic analyses were conducted using gonadal tissue as the experimental group and muscle tissue as the control. A total of 12,554 differentially expressed genes (DEGs) were identified (FDR < 0.05, |log2FC| > 1), including 9157 upregulated and 3397 downregulated genes. Gonadal development involves intensive cellular activities, including proliferation, gametogenesis, and hormone biosynthesis, all of which require coordinated activation of genes encoding metabolic enzymes, signaling molecules, and cell cycle regulators. Consequently, the number of upregulated genes far exceeded that of downregulated ones.

GO enrichment analysis revealed that DEGs were predominantly involved in cellular processes and metabolic activities. Molecular functions were enriched in binding and catalytic activities, suggesting that these DEGs play central roles in maintaining cellular functions and regulating metabolic states. The co-enrichment of terms related to “metabolic process” and “catalytic activity” implies that gene expression changes modulate energy metabolism alongside downstream signaling cascades. Moreover, the enrichment of protein complex components suggests a potential role for multiprotein assemblies in regulating these physiological processes.

KEGG pathway analysis indicated significant enrichment of DEGs in carbon metabolism (ko01200), glycolysis/gluconeogenesis (ko00010), and pyruvate metabolism (ko00620). Carbon metabolism serves as a central hub for generating energy and biomolecular precursors, such as amino acids, lipids, and nucleotides [106]. Glycolysis, occurring in the cytoplasm, converts glucose into pyruvate with the generation of ATP and NADH, functioning under both aerobic and anaerobic conditions to meet immediate energy demands [107]. Gluconeogenesis, by contrast, synthesizes glucose from non-carbohydrate sources, such as lactate, glycerol, and glucogenic amino acids, playing a critical role in maintaining energy homeostasis [108]. Pyruvate, the end product of glycolysis, can be further oxidized by pyruvate dehydrogenase (PDH) to form acetyl-CoA, which enters the tricarboxylic acid (TCA) cycle [109]. In total, 75 DEGs were associated with carbon metabolism (59 upregulated and 16 downregulated), 49 with glycolysis/gluconeogenesis (31 upregulated and 18 downregulated), and 29 with pyruvate metabolism (23 upregulated and 6 downregulated), with some DEGs enriched across multiple pathways. These findings indicate substantial differences in energy metabolism between gonadal and muscle tissues in *L. maculatus*.

To further explore the regulatory network, the DEGs enriched in these energy-related pathways were mapped to a protein–protein interaction (PPI) network. Sixteen core genes—GPI, got2a, GCSH, Taldo1, tpi1b, PGAM1, ENO1, AMT, SHMT2, GLDC, EON3, PAGM2, tpi1, eno4, BPGM, and Pgk1—were identified as potential key regulators of energy allocation between growth and reproduction. These genes provide promising targets for future investigations into the molecular mechanisms underlying energy metabolism in reproductive physiology.

#### 3.3.2. Analysis of Gonadal and Muscle Tissue-Targeted Energy Metabolism

The energy metabolism of the OFWLT and MFWLT groups showed that the ATP content of the OFWLT group was significantly higher than that of the MFWLT group (*p* < 0.05). In contrast, the ADP content in the OFWLT group was significantly lower than that in the MFWLT group (*p* < 0.05). Gonadal tissues (e.g., testes or ovaries) are primarily involved in activities such as hormone synthesis and germ cell development, both of which require a steady supply of energy. Studies have shown that steroid hormone synthesis depends on ATP supplied by mitochondria to support cholesterol transport, the catalytic reactions of steroidogenic enzymes, and the activation of enzyme systems in the endoplasmic reticulum [110]. Hormone synthesis and cellular secretory activities demand substantial energy, which is immediately provided by ATP. During hormone synthesis and secretion, ATP plays a central role in multiple cytosolic steps, including vesicle transport, docking, priming, and membrane fusion [111,112,113]. In addition, ATP not only provides energy for biosynthetic pathways but also regulates hormone release by maintaining intracellular calcium homeostasis [114]. Therefore, a continuous supply of ATP is essential for maintaining normal gonadal cell function and timely hormone secretion.

The higher ATP content of ovarian tissues may be related to their demand for intracellular synthetic reactions, such as hormone and protein synthesis. High ATP levels may reflect the energetic demands of hormone synthesis and germ cell development, as well as the capacity for rapid ATP generation via aerobic metabolism. Cells in gonadal tissues exhibit high proliferation and differentiation rates, especially during development and gametogenesis. These processes require large amounts of ATP to support DNA synthesis, protein synthesis, and cell division. During follicular development, granulosa cell proliferation and differentiation depend on mitochondrial ATP to support cell cycle progression and maintain cellular functions [115]. Oocytes also experience significantly increased energy demands during maturation, primarily met via oxidative phosphorylation for ATP production [116]. Similarly, male germ cell development, including spermatogonial differentiation, depends on ATP generated by mitochondrial respiration to support rapid proliferation and differentiation [117]. Together, these studies indicate that ATP is crucial for gonadal cell proliferation and differentiation, with high ATP levels reflecting rapid tissue growth and functional activity. The ATP/ADP ratio is an important indicator of cellular energy status, with a higher ratio signifying adequate energy availability [118]. Thus, significantly high ATP and low ADP in ovarian tissues imply an elevated ATP/ADP ratio, indicating that the gonads are in a state of high metabolic activity and can provide sufficient energy to support processes such as oocyte and sperm biosynthesis. In contrast, the relatively low ATP and high ADP in muscle tissue suggest that it may be in a state of energy mobilization or sacrifice to fuel gonadal development. It has been found that fish mobilize body wall tissues (such as muscle and fat) during spawning migration to supply energy for ovarian development. For example, in *Megalobrama terminalis*, lipid reserves in somatic tissues are significantly reduced during spawning migration to support gonadal development [28]. It has also been shown that the energy required for gonadal development is mainly derived from endogenous lipid and protein reserves stored in the liver and muscle tissues [119]. This phenomenon is consistent with the relative decrease in ATP and increase in ADP in the muscle of the *L. maculatus* in the present study, suggesting that the muscle tissue may redeploy energy to gonadal tissues. ATP, CA, and ICA were significantly higher (*p* < 0.05) than in the MFWLT group, whereas AKG, SA, and LA were significantly lower (*p* < 0.05) than in the MFWLT group. The content of PA was lower but not significantly different in the OFWLT group (*p* > 0.05). CA is a tricarboxylate formed by the condensation of acetyl coenzyme A and oxaloacetate, catalyzed by citrate synthase [120]. CA is isomerized to ICA by a two-step process involving dehydration and rehydration, catalyzed by aconitase, with cis-aconitate as the intermediate. The structural transition from CA to ICA is critical for the subsequent steps of the cycle, as it prepares the molecule for oxidative decarboxylation [121]. AKG plays a crucial role in the citric acid cycle as an intermediate in oxidative decarboxylation. The enzyme isocitrate dehydrogenase first dehydrogenates ICA to oxalosuccinate, which is then decarboxylated to AKG. AKG is a key precursor in the synthesis of amino acids, such as glutamate, which can be further converted to other amino acids and neurotransmitters [122]. CA can also be exported to the cytoplasm, where it is cleaved by ATP-citrate lyase to produce acetyl coenzyme A and oxaloacetate, which provide building blocks for fatty acid and cholesterol synthesis.

The large accumulation of early TCA cycle intermediates, such as CA and ICA, in gonadal tissues—accompanied by elevated ATP levels—suggests enhanced oxidative phosphorylation and increased TCA cycle flux to meet the energy demands of germ cell development and hormone synthesis. The reduced levels of AKG and SA, mid- and down-stream TCA intermediates, imply rapid consumption or diversion into biosynthetic pathways. This pattern—accumulation of upstream substrates (CA and ICA) coupled with depletion of downstream intermediates (SA)—is characteristic of increased TCA cycle flux and high-turnover aerobic metabolism. Moreover, because SA accumulation typically indicates hypoxia or circulatory blockade, the concurrent low SA and high ATP levels here further support a fully oxidized metabolic state [123].

By contrast, the elevated lactate content in muscle suggests a metabolic shift toward glycolysis, indicating anaerobic or oxygen-limited energy production. Studies in hybrid bass found that muscle converts glucose to pyruvate for the TCA cycle or to lactate for glucose recycling via the Cori cycle [124]. In *Salmo gairdneri* and *Cyprinus carpio*, increased activity of glycolytic enzymes in muscle supports rapid ATP generation via glycolysis during intense activity or hypoxia [125]. Overall, ovarian energy metabolism is driven primarily by oxidative phosphorylation and the TCA cycle, whereas muscle relies more on glycolysis.

Muscle lactate accumulation may fuel both local metabolism and serve as a substrate for gonadal energy via the lactate–glucose cycle. In *O. mykiss*, lactate serves as a major substrate for muscle glycogen synthesis [126]. The liver is the primary site of glycogen storage, whereas muscle contains lower glycogen levels [127]. Lactate or glucose produced by muscle may be metabolized and recirculated via the liver to meet systemic energy demands. In fish, gonadal development is accompanied by metabolic reprogramming; for example, spawning in *O. mykiss* induces a shift in muscle metabolism from glucose to lipids to support reproduction [128]. During spawning, *O. mykiss* exhibit upregulation of genes involved in mitochondrial ATP synthesis, fatty acid oxidation, and protein catabolism, concomitant with muscle mass reduction [129]. This suggests that muscle proteins and lipids are catabolized—sacrificing muscle growth—to supply amino acids and fatty acids for oxidative energy in support of gonadal development. During gonadal development, ovarian tissues enhance oxidative phosphorylation and TCA cycle activity to meet high reproductive energy demands. In contrast, muscle tissues rely on rapid glycolysis and may support ovarian energy needs via metabolites, such as lactate.

#### 3.3.3. Transcriptome and Targeted Energy Metabolome Association Analysis of Gonadal and Muscle Tissues

Correlation analysis of the transcriptome and targeted energy metabolome revealed upregulation of metabolites (CA, ICA, MA, and FA) and enzymes (*PC*, *ACO*, *IDH3*, *ACLY*, *SDHB*, and *OGDH*) in the mid-region of the TCA cycle in ovaries, indicating enhanced activity in this cycle segment. This likely facilitates the provision of intermediate metabolites for biosynthesis beyond energy metabolism [130]. Conversely, downregulation of *DLAT* and *DLD* indicates restricted pyruvate conversion to acetyl-CoA, whereas upregulation of *PC* suggests pyruvate enters the TCA cycle predominantly via carboxylation to oxaloacetate, favoring anabolism over oxidative metabolism [131]. This metabolic shift aligns with *ACLY* upregulation, implying mitochondrial CA export to the cytoplasm for cleavage by ACLY into acetyl-CoA and oxaloacetate, substrates for fatty acid synthesis and steroidogenesis [132]. ACLY catalyzes the cytoplasmic conversion of CA to acetyl-CoA, a key precursor in endogenous fatty acid and cholesterol biosynthesis [133]. Upregulated *IDH3* enhances AKG production; however, AKG levels decrease, likely reflecting rapid utilization for biosynthesis or oxidation rather than progression through the TCA cycle. This suggests that the gonadal TCA cycle favors supplying biosynthetic precursors and mitochondrial–cytoplasmic substrate transport over exclusive ATP production [134]. Glycolytic activity appears reduced, as evidenced by downregulation of key enzymes (*GPI*, *ALDO*, *GAPDH*, *PGK*, and *BPGM*) and corresponding decreases in downstream metabolites (PEP, PA, and LA). Conversely, upregulation of upstream genes (*PGM2* and *G6PC*) and increased D-glucose levels suggest enhanced glucose metabolism. This may maintain metabolic homeostasis or supply substrates and NADPH for ancillary pathways, such as the pentose phosphate pathway [135]. NADPH is essential for biosynthesis of fatty acids, cholesterol, and nucleic acids [136]. LDH downregulation correlates with reduced lactate production, consistent with oocytes relying on surrounding cells to supply LA or PA [137]. Overall, these findings indicate that ovarian gluconeogenesis prioritizes substrate conservation for anabolism rather than energy production. Mitochondrial electron transport chain (ETC) subunits—including *NDUFA* (complex I), *SDHB* (complex II), *QCR6* (complex III), *ATP4A*, and *ATPV1C* (complex V)—were upregulated in ovaries. Concurrently, ATP and flavin mononucleotide (FMN) levels increased, while NADH and ADP levels decreased, indicating enhanced ETC function to meet high ATP demands during oocytes’ proliferation, differentiation, and steroidogenesis. Elevated ATP and reduced ADP levels further support enhanced mitochondrial oxidative phosphorylation during ovarian development. This metabolic strategy provides a stable and continuous energy supply, aligning with the substantial energy requirements of oocytes during growth and development.

## 4. Materials and Methods

### 4.1. Ethics Statement

*L. maculatus* is not classified as an endangered or protected species in China, and there is no requirement for permission to undertake experiments in China.

### 4.2. Experimental Animals and Sample Collection

Two-year-old *L. maculatus*, obtained from Zhuhai Yueshun Farm in China and reared in brackish water (salinity: 4‰, mean weight: 1963 g), were used as experimental subjects. A total of 160 fish were divided into 4 groups of 40 fish each: Group 1: seawater normothermic group (salinity: 30‰, temperature: 30 ± 1 °C), referred to as SWNT; Group 2: seawater low-temperature group (salinity: 30‰, temperature: 18 ± 1 °C), referred to as SWLT; Group 3: brackish water low-temperature group (salinity: 4‰, temperature: 18 ± 1 °C), referred to as FWLT; Group 4: brackish water normothermic group (salinity: 4‰, temperature: 30 ± 1 °C), referred to as FWNT. These abbreviations were used for convenience in subsequent analyses. The fish were transported to the experimental aquaculture facility and acclimated in four 30 m^2^ circular tanks without feeding for seven days, and the salinity of the culture water is 4‰ and the temperature is 30 ± 1 °C. After acclimation, the SWNT and SWLT groups underwent a salinity increase of approximately 10‰ per day for 3 consecutive days until reaching 30‰, while salinity in the FWLT and FWNT groups was maintained at 4‰. Following salinity adjustment, the SWLT and FWLT groups were subjected to temperature reduction at a rate of 2–3 °C per day for 4 consecutive days until reaching 18 ± 1 °C. The SWNT and FWNT groups remained at 30 ± 1 °C. The experiment officially began one week after the salinity and temperature of the water had been adjusted. The experiment lasted for six weeks. Feeding was initiated using floating pellets for acclimation. Once acclimated, the diet transitioned to a combination of pellet feed and chilled bait. Feeding amounts were adjusted based on appetite and maintained at 1–2% of body weight. One-third of the tank water was replaced daily throughout the experiment. Two samplings were conducted during the experiment. The first sampling session was completed on the first day of the first week of the experiment, with five female fish randomly selected from each of the SWNT, SWLT, FWLT, and FWNT groups (the experimental fish were a mixed population of males and females, which were distinguished by their gonads after post-dissection). Each fish was weighed, and gonad weight was recorded using a precision electronic balance to calculate the gonadosomatic index (GSI). Blood samples were collected from the caudal vein using 5 mL syringes, centrifuged at 3000 rpm for 10 min, and the serum was stored at −20 °C for subsequent hormone analysis. Muscle and ovarian tissues were collected and immediately frozen in liquid nitrogen for transcriptomic and metabolomic analysis. The second sampling was conducted on the last day of the sixth week of the experiment following the same procedures.

### 4.3. Calculation of Gonadal Index

The gonadal index was used to determine the degree of ovarian development based on the data recorded for each group of fish at the two sampling times. Gonadal index = (gonadal weight/body weight) × 100%.

### 4.4. Detection of Reproduction-Related Hormones

Serum levels of estradiol (E2), luteinizing hormone (LH), follicle-stimulating hormone (FSH), and 17α,20β-dihydroxyprogesterone (17α,20β-DHP) were measured using an enzyme-linked immunosorbent assay (ELISA) with a commercial hormone detection kit. The hormone assay kit was supplied by Huangshi Aynes Biotechnology. Frozen serum samples were thawed at room temperature for 10–30 min, with periodic checks to confirm complete liquefaction. The thawed samples were inverted to check for the presence of flocculent material. If flocculation was observed, the samples were centrifuged at 3000 rpm for 10 min, and the supernatant was collected for analysis. The assay was conducted strictly following the manufacturer’s instructions. The standard concentration was plotted on the x-axis and the optical density (OD) value on the y-axis. Hormone concentrations were calculated using ELISA Calc software (v 0.1) and multiplied by the dilution factor to determine the final serum hormone levels.

### 4.5. RNA Extraction and Transcriptome Analysis

Three ovarian tissue samples from the SWNT group, SWLT group, FWNT group, and FWLT group, and three muscle tissue samples from the FWLT group, were randomly selected from the second sampling as biological replicates for transcriptomic analysis. Total RNA was extracted using the Trizol method. Samples were analyzed for RNA integrity and DNA contamination using agarose gel electrophoresis. RNA purity was assessed using a NanoDrop 2000, with OD260/280 values between 1.9 and 2.1 and OD260/230 values greater than 2.0 considered acceptable. RNA integrity was further confirmed using an Agilent 2100 Bioanalyzer. Sequencing libraries were constructed using the TruSeq Stranded mRNA LT Sample Prep Kit (Illumina, San Diego, CA, USA) and sequenced using the Illumina HiSeq™2500 platform (Illumina, San Diego, CA, USA). Raw sequencing data were filtered to obtain clean reads, which were then aligned to the *L. maculatus* genome database to quantify gene expression levels in each sample. Gene expression levels were represented by both raw read counts and fragments per kilobase of transcript per million mapped reads (FPKMs). Principal component analysis (PCA) was performed using R “http://www.r-project.org/ (accessed on 10 January 2025) to evaluate intra-group consistency and inter-group variability. Read counts were normalized using the edgeR package (R 4.0), and *p*-values were calculated based on the statistical model. Multiple testing correction was applied to calculate the false discovery rate (FDR). Differential gene expression was visualized using bar charts and volcano plots, with FDR < 0.05 and |log2FC| > 1 as the thresholds for significance. Hierarchical clustering of differentially expressed genes was performed using log10-transformed FPKM values, and the results were visualized as heatmaps. Differentially expressed genes were subjected to functional and pathway enrichment analyses using the GO (http://www.geneontology.org/) (accessed on 10 January 2025) and KEGG (http://www.genome.jp/kegg/) (accessed on 10 January 2025) databases.

### 4.6. RT-qPCR Validation of Differentially Expressed Genes

Real-time quantitative reverse transcription PCR (RT-qPCR) was used to analyze and validate the transcriptional expression levels of eight differentially expressed genes (DEGs). Gene-specific primers were designed using Primer5 (Premier, Charlotte, NC, USA), with the β-actin gene used as an internal reference (primer sequences are listed in Table 2). Three biological and three technical replicates were performed. RT-qPCR was performed using the FastReal Rapid Fluorescence Quantitative PCR Kit (Tiangen, Beijing, China). Amplification was conducted in a 10 μL reaction volume, which included 5 μL of 2× FastReal qPCR PreMix, 0.3 μL each of forward and reverse primers (10 μmol/L), 3.4 μL of ddH_2_O, and 1 μL of cDNA template. A two-step cycling method was followed according to the manufacturer’s instructions. The thermal cycling conditions included pre-denaturation at 95 °C for 3 min, denaturation at 95 °C for 5 s, annealing at 60 °C for 15 s, and extension at 72 °C for 15 s. The PCR program was run for 40 cycles. A melt curve analysis confirmed that each PCR product produced a single peak. The data were analyzed using the 2^−ΔΔCT^ method.

### 4.7. Targeted Energy Metabolomics Analysis

Five ovarian and muscle tissue samples were randomly selected from the second samplings of the FWLT group. An appropriate amount of each sample was weighed and placed into a 2 mL centrifuge tube, followed by the addition of 500 μL of tissue extract (75% methanol: 25% H_2_O) and two steel beads. The samples were ground using a cryo-mill at 60 Hz for 1 min. After grinding, they were shaken using a thermostatic metal shaker at 1500 rpm and 4 °C for 10 min, then centrifuged at 4 °C and 12,000 rpm for 10 min. Two 100 μL aliquots of the supernatant were pipetted into two new 2 mL centrifuge tubes. An appropriate amount of mixed internal standard was added to each tube, followed by thorough mixing, concentration, and drying. In one dried tube, 50% aqueous acetonitrile was added to dissolve the extract, serving as the alkaline solution. In the other dried tube, 0.1% formic acid in 40% aqueous methanol was added to dissolve the extract, serving as the acidic solution. The resulting supernatants were transferred to injection vials for LC-MS analysis. Chromatographically separated effluent components were continuously introduced into the mass spectrometer for data acquisition via continuous scanning. Calibration curves were constructed using the standard concentration on the x-axis and the peak area ratio of the standard to the internal standard on the y-axis. Linear regression equations with correlation coefficients (r > 0.99) were obtained for each compound. Quantitative analysis for all samples was performed based on the established metabolite calibration curves. Raw data were converted from “.wiff” to “.mzML” format using ProteoWizard (v3.0.8789). Data were analyzed both qualitatively and quantitatively using SCIEXOS software (v3.4.5) (SCIEX, Shanghai, China). Orthogonal projections to latent structures discriminant analysis (OPLS-DA) was performed using the R package “ropls” (R 4.0) to reduce model complexity and enhance explanatory power without compromising predictive performance. OPLS-DA score plots were generated to assess inter-group and intra-group differences. Permutation test plots were used to evaluate the reliability of the model. Heatmaps were generated to visualize changes in energy-metabolism-related metabolites among groups. Significant metabolite differences were identified based on t-test *p*-values from the OPLS-DA model and fold change analysis. Pathway enrichment maps of differential metabolites were constructed and analyzed using the KEGG Mapper visualization tool (Panomix, Suzhou, China).

### 4.8. Combined Transcriptome and Energy Metabolome Analysis

Transcriptome and targeted energy metabolome association analysis was performed on ovarian and muscle tissues from the FWLT group. The analysis focused on genes and metabolites involved in the same metabolic pathways, based on KEGG pathway maps (www.kegg.jp). Genes and metabolites were represented using different marker shapes and fill colors. Red circles indicated upregulated metabolites, blue circles indicated downregulated metabolites, and white circles represented metabolites not detected in this study. Yellow squares represented upregulated genes, green squares indicated downregulated genes, and white squares represented genes with no or minimal changes. The results are displayed in Figure 8.

### 4.9. Statistical Analysis

Data were expressed as mean ± standard deviation (SD). Statistical analysis was performed based on SPSS 25.0, and plotting was done by GraphPad prism 9.5. Comparisons between two groups were made using t-tests, while comparisons between more than two groups were determined using one-way analysis of variance (ANOVA) and post hoc Tukey HSD tests. For all statistical comparisons, *p* < 0.05 was considered a significant difference between the two groups.

## 5. Conclusions

In this study, four treatment groups were established—SWNT (30‰, 30 ± 1 °C), SWLT (30‰, 18 ± 1 °C), FWLT (4‰, 18 ± 1 °C), and FWNT (4‰, 30 ± 1 °C). Only the FWLT group showed a significant increase in the gonadosomatic index (GSI) of *L. maculatus*, indicating a synergistic effect of low salinity (4‰) and low temperature (18 ± 1 °C) on ovarian development. Measurement of reproduction-related hormones revealed that FSH, E2, LH, and 17α,20β-DHP were significantly elevated in the FWLT group, indicating activation of the hypothalamic–pituitary–gonadal (HPG) axis. This enhanced signaling accelerated oocyte transition from early to mature stages, highlighting environmental regulation of endocrine function. Transcriptome analyses confirmed that temperature was the main driver of ovarian development in *L. maculatus*, whereas salinity induced fewer DEGs. Transcriptome and targeted energy metabolome correlation analyses showed that the expression of glycolysis-related enzymes was downregulated and glycolytic intermediates were reduced in the ovary, while the expression of oxidative-phosphorylation-related genes was upregulated, suggesting that the gonads favor aerobic metabolism for energy supply. Muscle is mainly supplied by glycolysis for rapid energy production, and it may redeploy energy to gonadal tissues to prioritize the energy demand of the ovary to balance the energy demand for somatic growth and reproduction and achieve a dynamic balance between reproduction and growth. Taken together, these findings deepen our understanding of how temperature and salinity interact to regulate fish reproductive development and shed light on energy allocation strategies between growth and reproduction during the reproductive phase of fish.

## Figures and Tables

**Table 1:** Transcriptome sequencing data.

Sample	Raw Data	Clean Data (%)	BF_Q30 (%)	BF_GC (%)
OSWNT1	8,444,406,300	832,965,228 (98.64%)	7,787,987,434 (92.23%)	4,307,011,897 (51.00%)
OSWNT2	7,264,622,400	719,390,209 (99.03%)	6,689,383,501 (92.08%)	3,687,134,980 (50.75%)
OSWNT3	7,129,401,000	705,316,141 (98.93%)	6,580,108,021(92.30%)	3,638,707,983 (51.04%)
OSWLT1	7,944,971,100	778,703,561 (98.01%)	7,413,924,316 (93.32%)	3,894,264,316 (49.02%)
OSWLT2	7,986,083,700	783,286,290 (98.08%)	7,441,185,395 (93.18%)	4,081,109,505 (51.10%)
OSWLT3	8,026,868,700	786,990,444 (98.04%)	7,487,352,558(93.28%)	4,026,903,542 (50.17%)
OFWLT1	7,953,954,300	786,547,287 (98.89%)	7,343,776,104 (92.33%)	3,783,132,177 (47.56%)
OFWLT2	7,734,025,200	762,521,171 (98.59%)	7,132,487,673 (92.22%)	3,891,404,658 (50.32%)
OFWLT3	8,354,551,200	825,821,772 (98.85%)	7,735,196,526 (92.59%)	4,032,074,641 (48.26%)
OFWNT1	8,932,483,500	882,958,414 (98.85%)	8,264,811,490(92.53%)	4,508,584,371 (50.47%)
OFWNT2	7,383,731,700	727,708,069 (98.56%)	6,809,242,523(92.22%)	3,755,759,027 (50.87%)
OFWNT3	7,595,829,600	7,367,262,628 (96.99%)	6,995,732,246 (92.10%)	3,874,770,036 (51.01%)

**Table 2:** Primer sequences.

Primer Name	Sequence (5′→3′)
Cpeb4-F	TATGAGCCGCAGCGACAG
Cpeb4-R	AGAGGCCCATCTCCACCCT
PRPS2-F	GGACATGCCTAACATCGTGC
PRPS2-R	CTCCTGGTTGCTGAACTTCTTG
ADCY6-F	TTACAATCTGTGAGCGGAAACT
ADCY6-R	CCATGTGCCAAATACAACCAT
MAT2A-F	GCAGCAGTCTCCTGACATCG
MAT2A-R	CATCAGTGGCATATCCAAACA
fzd7-a-F	ACTCCCTTTACTCCCACTTGA
fzd7-a-R	CCTACTGGTGTTTCCCTGATT
mapk12-F	TCAAAGCCATGCCGTAAGC
mapk12-R	GTCACCGAAAGCCACTCCC
pvalb2-F	GCCGACTCTTTTCAAGCACAA
pvalb2-R	CCTCCTCAATGAAGCCACTC
PYGM-F	GCCAGGAGAAAGTCAATGCT
PYGM-R	TACTCGGTAATGGGTGCGATC
GPI-F	ACCGCTTCACTGCCTACTTC
GPI-F	GTGGTCTTGCCCGTCATC
got2aR	CAAGCCTGAGGAGTGGAAGG
got2aR	CAAGCCTGAGGAGTGGAAGG
GCSH-1-F	CCGCTCCCTGTCTTCCAACT
GCSH-1-F	GTTCCCACCTCTGGCAGTCC
tpi1b-F	CAGCCAGTCCACGCCTTTA
tpi1b-R	CCTTCCCACCCTTCTACCC
PGAM1-1-F	GCCAAGAAGTAAAGCAACAGAA
PGAM1-1-F	AACATCCCAGTCGTTCACCA
AMT-R	GTCACATCGCCTCGCACAT
AMT-R	GGTCATCAATAATCCCTCCTTTCTC
ENO1-F	CCCACGCAGGCAACAAG
ENO1-R	AAGCCGCCCTCATCTCC
BPGM-F	AGGCTATGGGTCAGGAGTGG
BPGM-R	CCAGGAAGTAAGGGTGGGAT
β-actin-F	CAACTGGGGATGACATGGGGAGAAG
β-actin-R	TTGGCTTTGGGGGGTTCAGG

**Table 3:** Transcriptome sequencing data.

Sample	Raw Data	Clean Data (%)	BF_Q30 (%)	BF_GC (%)
OFWLT1	7,953,954,300	7,865,472,876 (98.89%)	7,003,646,337 (93.09%)	3,811,150,486 (50.66%)
OFWLT2	7,734,025,200	7,625,211,711 (98.59%)	5,747,382,251 (92.94%)	3,232,272,528 (52.27%)
OFWLT3	8,354,551,200	8,258,217,726 (98.85%)	7,072,551,701 (92.49%)	3,993,848,545 (52.23%)
MFWLT1	7,523,556,600	7,454,735,551 (99.09%)	7,343,776,104 (92.33%)	3,783,132,177 (47.56%)
MFWLT2	6,184,004,400	6,118,434,737 (98.94%)	7,132,487,673 (92.22%)	3,891,404,658 (50.32%)
MFWLT3	7,647,044,700	7,576,414,880 (99.08%)	7,735,196,526 (92.59%)	4,032,074,641 (48.26%)

## Data Availability

Data are contained within the article.

## Abbreviations

The following abbreviations are used in this manuscript:

*Lateolabrax maculatus*	*L. maculatus*
Gonadosomatic index	GSI
Estradiol	E2
Luteinizing hormone	LH
Follicle-stimulating hormone	FSH
17α,20β-dihydroxyprogesterone	17α,20β-DHP
Gonadotropin-releasing hormone	GnRH
Real-time quantitative reverse transcription PCR	RT-qPCR
Isocitrate	ICA
Cis aconitic acid	CAA
Citric acid	CA
Malic acid	MA
Fumaric acid	FA
Succinic acid	SA
Pyruvic acid	PA
α-Ketoglutarate	AKG
Flavin mononucleotide	FMN
Beta-D-fructose 1,6-bisphosphate	BDFP
Final oocyte maturation	FOM
